# Unveiling the multifaceted antitumor effects of interleukin 33

**DOI:** 10.3389/fimmu.2024.1425282

**Published:** 2024-05-31

**Authors:** Leire Arrizabalaga, Aline Risson, Miriam Ezcurra-Hualde, Fernando Aranda, Pedro Berraondo

**Affiliations:** ^1^ Cima Universidad de Navarra, Pamplona, Spain; ^2^ Navarra Institute for Health Research (IDISNA) and Cancer Center Clínica Universidad de Navarra (CCUN), Pamplona, Spain; ^3^ Centro de Investigación Biomédica en Red de Cáncer (CIBERONC), Madrid, Spain

**Keywords:** IL-33, cancer immunotherapy, tumor microenvironment, immune modulation, engineered cytokines

## Abstract

Interleukin 33 (IL-33), once predominantly recognized for its pro-tumoral activities, has emerged as a multifunctional cytokine with antitumor properties. IL-33 pleiotropic activities include activation of Th1 CD4^+^ T cells, CD8^+^ T cells, NK cells, dendritic cells, eosinophils, as well as type 2 innate lymphoid cells. Regarding this immunomodulatory activity, IL-33 demonstrates synergistic interactions with various cancer therapies, including immune checkpoint blockade and chemotherapy. Combinatorial treatments leveraging IL-33 exhibit enhanced antitumor efficacy across different tumor models, promising novel avenues for cancer therapy. Despite its antitumor effects, the complex interplay of IL-33 within the tumor microenvironment underscores the need for further investigation. Understanding the mechanisms underlying IL-33’s dual role as both a promoter and inhibitor of tumor progression is essential for refining therapeutic strategies and fully realizing its potential in cancer immunotherapy. This review delves into the intricate landscape of IL-33 effects within the tumor microenvironment, highlighting its pivotal role in orchestrating immune responses against cancer.

## 1 Introduction

Interleukin 33 (IL-33), initially identified as a nuclear factor in high endothelial venules (NF-HEV) (1), is structurally related to the cytokines of the IL-1 family and it is expressed by fibroblasts, endothelial and epithelial cells, among other cell types (2). The full-length human IL-33 precursor encompasses 270 amino acids, featuring an N-terminal region that includes a chromatin binding motif and a nuclear localization signal (amino acids 1–65), a sensor domain from amino acid 66 to 111, and a C-terminal IL-1-like cytokine domain (amino acids 112–270) that gives IL-33 its cytokine-like properties (3). The N-terminal region is formed by a homeodomain-like helix–turn–helix motif of 65 amino acids. The nuclear localization signal is located within amino acids 46–67 (4). The binding capacity to DNA determines the activities of full-length IL-33 in the nucleus as a transcription factor (5). Human and mouse IL-33 share 52% similarity at the amino acid level. Recombinant human IL-33 is as effective as recombinant mouse IL-33 in activating mouse lymphoid cells (6).

**Figure 1:**
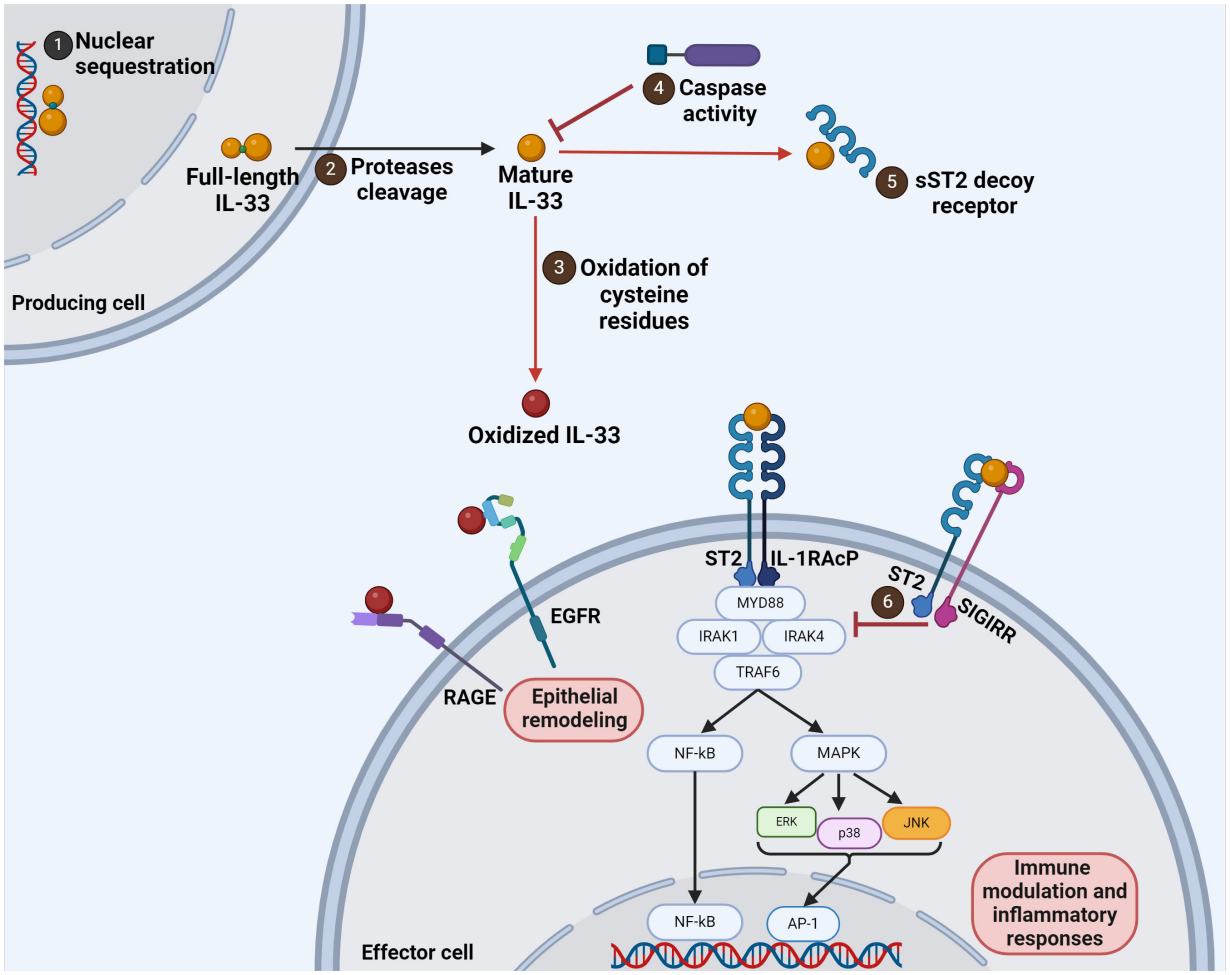
Interleukin 33 regulatory mechanisms and signaling pathways. The full-length IL-33 molecule of 33 kDa can be found in the nucleus and retained there (1). When cellular injury occurs, IL-33 is released and processed to a mature form by protease cleavage (2). The mature form of IL-33 has a high affinity to the ST2 receptor. This mature IL-33 can be modified by oxidation, which generates an oxidized form that mediates epithelial remodeling when recognized by RAGE and EGFR (3). However, the mature IL-33 can also be inactivated by caspases (4), a decoy receptor formed by a soluble form of ST2 (5), or by SIGIRR binding (6).

The IL-33 gene, situated on chromosome 9 (9p24.1) in humans and in mice, located at 19qC1 on the same chromosome (3), encodes the precursor protein for IL-33, known as full-length IL-33. Transcriptional regulation of the IL-33 gene is subject to various factors, including inflammatory stimuli, tissue damage, and cellular stress. Notably, epigenetic modifications, such as DNA methylation and histone acetylation, play crucial roles in modulating IL-33 expression, thereby influencing its physiological functions and pathological implications. Additionally, transcription factors, such as NF-κB (nuclear factor kappa-light-chain-enhancer of activated B cells) and AP-1 (activator protein 1), are implicated in the regulation of IL-33 gene expression, orchestrating its responsiveness to diverse extracellular cues and intracellular signaling cascades. In the steady state, full-length IL-33 is constitutively expressed, and upon cellular damage or necrosis, the expression of IL-33 is upregulated, and the full-length IL-33 is processed to mature IL-33 (7). Additionally, immune homeostasis depends on nuclear localization and sequestration of the full-length IL-33, which control IL-33’s release and pro-inflammatory effects (**Figure 1**) (3).

After cellular injury, the 33 kDa full-length IL-33 can be cleaved in the sensor domain by several endogenous or exogenous proteases (elastase, cathepsin G, chymase, tryptase, granzyme B) (8) to generate a 19 kDa version of the protein defined as the mature form (IL-33_95-270_, IL-33_99-270_, and IL-33_109-270_), with 10 to 30 times higher biological activity (9). This form of IL-33 elicits immune responses via its interactions with specific receptors, acting as an alarmin (10).

IL-33 exerts mainly its pro-inflammatory effects by engaging with specific cell surface receptors. The main receptor is composed of the ST2 (suppression of tumorigenicity 2) receptor (also known as IL-1 receptor-like 1 IL-1RL1, T1, DER4, or Fit-1) in association with the IL-1 receptor accessory protein (IL-1RAcP). The ST2 receptor exists in two isoforms: a membrane-bound form (ST2L) and a soluble form (sST2). While ST2L mediates the pro-inflammatory signaling cascades triggered by IL-33, sST2 acts as a decoy receptor, antagonizing IL-33-induced responses (11). Moreover, the pro-inflammatory activity of IL-33 is also regulated by oxidation. The four cysteines of the protein sequence can generate disulfide bridges, resulting in an extensive conformational change that disrupts the binding to the receptor ST2. Alternatively, IL-33 can be rendered inactive by proteolytic cleavage by caspases (5). The caspase cleavage site is located at amino acid 178 in the IL-1 cytokine domain and is not present in other members of the IL-1 superfamily (3). The inactivation of the IL-33/ST2 axis can also be mediated by the negative regulator SIGIRR (single immunoglobulin IL-1R-related molecule). It has been suggested that SIGIRR binds to ST2 in a complex formed upon IL-33 activation, restricting the recruitment of the IL-1RAcP adaptor protein (9).

Following ST2 receptor binding, IL-33 initiates intracellular signaling pathways, culminating in the activation of various downstream effectors involved in immune modulation and inflammatory responses (12). The canonical signaling cascade triggered by IL-33 involves the recruitment of MyD88 (myeloid differentiation primary response 88), IRAK (IL-1 receptor-associated kinase) 1 and 4, and TRAF6 (tumor necrosis factor receptor-associated factor 6), leading to the activation of NF-κB and MAPK (mitogen-activated protein kinase) signaling pathways. Subsequently, these signaling events orchestrate the transcriptional regulation of target genes associated with inflammation, tissue repair, and immune cell activation (**Figure 1**) (13). ST2-independent activities of IL-33 have been described, pointing to an unidentified receptor associating with IL-1RAcP (13, 14). Moreover, the oxidized forms of IL-33 bind to the receptor for advanced glycation end products (RAGE) and epidermal growth factor receptor (EGFR), providing another alternative pathway to ST2. Following ligand binding, RAGE activates downstream signaling pathways involving AP-1, NFAT, NF-κB, STAT3, and CREB transcription factors. Oxidized IL-33 leads to phosphorylation of signaling molecules (JNK, ERK1/2, and STAT5) downstream of EGFR, inducing a reduction in epithelial defense functions (15, 16). Tozorakimab (MEDI3506), a strong human anti-IL-33 monoclonal antibody, has recently been shown to prevent IL-33 from oxidizing and from acting through the RAGE/EGFR signaling pathway, causing a reduction in inflammation and epithelial dysfunction (17).

## 2 Pleiotropic antitumor activity of IL-33

**Figure 2:**
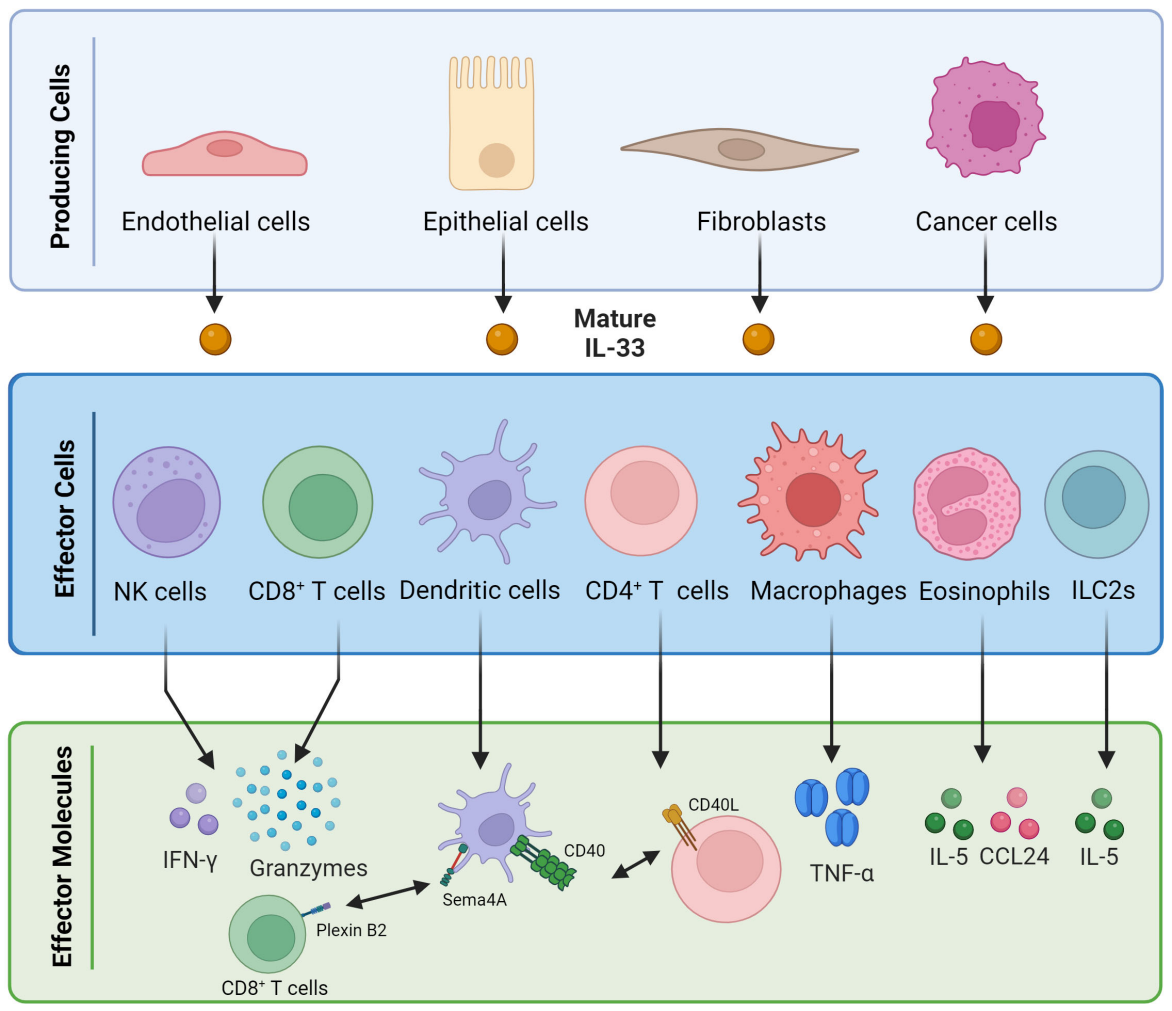
Pleiotropic immune activities of interleukin 33. Mature IL-33 can be released by various types of cells, including endothelial cells, epithelial cells, fibroblasts, and cancer cells. Once released, it can influence the activity of different types of immune cells, such as NK cells, CD8^+^ T cells, dendritic cells, CD4^+^ T cells, macrophages, eosinophils, and innate lymphoid cells, inducing the release of several soluble factors.

Despite the well-established pro-tumor activity of IL-33, this cytokine has been shown to potentiate Th1-mediated immune responses, the most relevant in tackling cancer. IL-33 synergizes with interleukin 12 (IL-12) to promote Th1 CD4^+^ T cells and the production of IFN-γ by CD8^+^ T lymphocytes and natural killer (NK) cells (18, 19). The ST2 receptor is not expressed in NK cells at baseline but is upregulated upon exposure to IL-12 or TNF-α (19, 20). In CD8^+^ T lymphocytes, the expression of ST2 is dependent on the transcription factor T-bet, expressed in effector CD8^+^ T cells (18). This capacity of IL-33 plays a pivotal role in shaping the tumor microenvironment (TME) and influencing tumor growth. Overexpression of IL-33 in B16 melanoma cells or 4T1 breast cancer cells highlighted the effect of IL-33 as a potent inhibitor of tumor progression, exerting its effects through the modulation of CD8^+^ T and NK cells. Notably, IL-33 not only promotes the functionality of CD8^+^ T cells but also induces robust type 1 antitumor immune responses. This induction leads to an upsurge in the production of critical type 1 effector molecules, including IFN-γ and granzymes, by both CD8^+^ T and NK cells within TME (**Figure 2**). As tumors grow, they trigger inflammatory responses and express tumor antigens, setting the stage for the dynamic interplay between the immune system and malignancy. Tumor progression, however, faces resistance from type 1 lymphocytes (Th1 CD4^+^ T cells, CD8^+^ T cells, NK cells, and γδ T cells) and collaborating innate cells (dendritic cells, M1 macrophages, and N1 neutrophils). Despite these defensive mechanisms, tumors manage to advance by recruiting immune suppressive cell types like T regulatory cells (Treg) and myeloid-derived suppressor cells (MDSC) (21).

In addition to directly affecting T lymphocytes and NK cells, IL-33 orchestrates the antitumor immune response acting on dendritic cells. Dendritic cells (DCs) are critical for initiating effective cancer immune responses. In these cells, IL-33 induces the expression of semaphorin 4A (Sema4A) that regulates CD8^+^ T cell activation through the receptor plexin B2 (22, 23). Interestingly, the antitumor activity of recombinant IL-33 (rIL-33) in the Lewis lung carcinoma (LLC) model was totally abrogated in the Sema4A knock-out (KO) mouse, indicating the relevance of the activation of DCs in the IL-33-mediated antitumor activity (22). An alternative mechanism for activating DCs is the upregulation of CD40L on CD4^+^ T lymphocytes. CD40L binds to CD40 expressed on DCs, triggering the maturation and activation of these cells. Activated DCs exhibit enhanced antigen-presenting capabilities, upregulating the expression of co-stimulatory molecules and cytokines. This, in turn, facilitates the activation and differentiation of T cells. Interestingly, it has been demonstrated that IL-33 is a potent inductor of CD40L on CD4^+^ T lymphocytes (24).

By using ST2 KO mice, several groups have demonstrated the essential role of this receptor for the antitumor activity of IL-33 in different tumor models (20, 24, 25). Another key mediator of the IL-33 activity located downstream from this receptor is MyD88. This is a common adaptor utilized by many Toll-like receptors (TLRs) and interleukin 1 receptors (IL-1Rs), including the IL-33 receptor complex. Upon binding of IL-33 to ST2, MyD88 is recruited to the cytoplasmic domain of ST2. MyD88 acts as a bridge, connecting the receptor complex to downstream signaling components. Interestingly, the profound impact of rIL-33 on tumor immunity in the LLC lung cancer model was not replicated in MyD88^-/-^ mice, underscoring the crucial role of the MyD88 pathway in mediating IL-33’s immunomodulatory effects. Further insight into the mechanism revealed that IL-33 required the MyD88 pathway to promote the activation of CD8^+^ T, NK cells, and DCs (26).

In certain tumor indications, other immune cell populations have been described as important mediators for the antitumor effect of IL-33. In the case of pulmonary metastasis of 4T1 breast cancer or B16 melanoma tumors, macrophages are the main mediator of the antitumor activity of rIL-33. These cells produce large amounts of TNF-α upon IL-33 inoculation. TNF-α induces the expression of ST2 in NK cells, sensitizing this effector immune effect to the activity of rIL-33 (20). In a pancreatic ductal adenocarcinoma (PDA) mouse model, Dixit A. et al. demonstrated that blocking the infiltration of bone marrow-derived macrophages through various CCR2 inhibition strategies significantly reduces TNF-α levels and elevates IL-33 levels. Interestingly, the earlier discovery that PDA patients with higher IL-33 levels have longer survival is supported by the increased presence of CD103^+^ dendritic cells and CD8^+^ cytotoxic T cells. This increase is attributed to the elevated IL-33 resulting from the reduction of immunosuppressive myeloid cells and TNF-α (27).

Another important immune cell population regarding IL-33 antitumor activity is eosinophils. In CT26 colon cancer cell engraftment models, IL-33 treatment demonstrated a notable reduction in tumor growth, a phenomenon intricately linked to eosinophils. This dependency was elucidated as eosinophil-deficient mice exhibited an absence of the growth reduction effect, which was subsequently restored upon the adoptive transfer of ex vivo-activated eosinophils. Mechanistically, IL-33 emerged as an eosinophil regulator, amplifying the expression of activation and homing markers (CD11b and Siglec-F) and degranulation markers (CD63 and CD107a). Moreover, IL-33 not only enhanced the viability, cytotoxic potential, and migration properties of eosinophils but also contributed to the heightened levels of IL-5 and CCL24 in tumors (**Figure 2**) (28). Blomberg et al. described that the patients with metastatic triple-negative breast cancer who responded to immune checkpoint blockade (ICB) therapy had increased eosinophils levels, and the accumulation of these cells was induced by CD4^+^ T cells, IL-5, and IL-33 (29).

Eosinophils and macrophages also participate in a multipronged immune response orchestrated by IL-33 to eradicate tumors in models of peritoneal carcinomatosis. Thus, depletion of CD4^+^ T cells, eosinophils, or macrophages significantly diminishes the benefits conferred by IL-33, highlighting the intricate interplay between these immune components and the cytokine’s protective effects. Surprisingly, the absence of B cells during IL-33 treatment takes an unexpected turn, as it leads to an increase in overall survival rates (30). Another report supported the relevant role of IL-33 in shaping an antitumor response in the peritoneal compartment. Overexpression of IL-33 emerged as a pivotal factor in prolonging survival and favoring the elimination of tumors in the peritoneum, but not when the ID8 ovarian cancer cell line was implanted subcutaneously. A crucial aspect of IL-33’s impact lies in its ability to inhibit MDSC differentiation, thereby promoting a robust tumor immune response within the ascitic environment (31). It has also been suggested that type 2 innate lymphoid cells (ILC2s) mediate the recruitment of eosinophils through IL-5 production in the case of melanoma models based on intravenous inoculation of B16.F10 cells to generate lung metastases (32) or subcutaneously implanted B16-F10 tumors (33). These cells express the ST2 receptor, and IL-33 is a potent factor that induces their proliferation and activation (34).

## 3 Combinatorial treatments encompassing IL-33

The combination of IL-33 and ICB represents a compelling strategy, as evidenced by multiple studies. The induction of IL-33 in tumor cells following treatment with ICB, such as cytotoxic T-lymphocyte antigen-4 (CTLA-4) and programmed death-1 (PD-1) monoclonal antibodies (mAbs), establishes a significant link between ICB immunotherapy and IL-33 expression within the TME (25).

One key aspect contributing to the effectiveness of ICB immunotherapy is the ST2 signaling in non-tumor cells, particularly CD8^+^ T cells. IL-33 plays a crucial role by augmenting the accumulation and effector function of tumor-resident CD103^+^ CD8^+^ T cells. Additionally, IL-33 contributes to increased numbers of CD103^+^ DCs in the TME, which are vital for both the antitumor effect of IL-33 and the accumulation of tumor-infiltrating CD8^+^ T cells (25). In pancreatic cancer, IL-33 has been shown to mediate the activation of ILC2s, which anti-PD-1 mAbs can further expand. These cells emerge as a potential strategy to enhance T cell priming within cancers infiltrated by ILC2s (21).

The key effect of endogenous IL-33 on the antitumor efficacy of ICB can be enhanced by exogenous administration of IL-33. This has been demonstrated through the overexpression of IL-33 by tumor cells that enhanced the antitumor activity of anti-CTLA-4 mAb or anti-PD-1 mAb in a B16 melanoma model (25, 35). Moreover, rIL-33 also enhanced the antitumor activity of anti-CTLA-4 mAb in a model of 4T1.2 lung metastases (16) or of anti-PD-1 mAb in a mouse model of pancreatic adenocarcinoma (36). In addition to this, intraperitoneal administration of IL-33 was combined with an anti-CSF1R or p38 inhibitor to treat peritoneal metastasis of gastric cancer. This promising approach prevents M2 polarization of macrophages by blocking the activation of the p38-GATA3 signaling pathway (37).

IL-33 synergy has also been demonstrated with chemotherapy. In colorectal cancer (CRC), the role of IL-33 emerges as a crucial factor in influencing the efficacy of 5-fluorouracil (5-FU) treatment. The presence of IL-33 not only enhances the sensitivity of CRC cells to 5-FU but also plays a pivotal role in creating an immune-active TME. This occurs through the orchestration of antitumoral T-cell responses, where IL-33 acts as a key mediator in activating T cells (38).

The field of adoptive cell transfer in cancer immunotherapy is witnessing promising advancements, and the ability of IL-33 to generate an antitumor immune response can be used to potentiate tumor-specific T cells. It has been evaluated the antitumor effect of chimeric antigen receptor T cells (CAR T) engineered to express the interleukin 2 (IL-2) superkine “Super2” and IL-33. Super2 is a variant of human IL-2 designed to bind to the intermediate IL-2 receptor complex expressed on naïve T cells and NK cells with 200-fold higher affinity than native IL-2. Super2 and IL-33 armored CAR T cells can reshape the TME, recruiting and activating a broad repertoire of immune cells, and promote antitumor immunity in multiple solid tumor models (39). A similar strategy was pursued by George Koukos and colleagues (40). OT1 ovalbumin-specific T cells were used as tumor-specific T cells, and these cells were engineered to express an IL-2 variant (IL-2v), which does not bind to the high-affinity IL-2 receptor α-chain (CD25), and IL-33. IL-2v was selected to promote CD8^+^ T-cell stemness, while IL-33 was introduced to potentiate the cross-priming potential of tumor-associated dendritic cells. The combination generated a unique effector state in the transferred T cells characterized by high expression of granzymes and downregulation of multiple transcription factors associated with T-cell dysfunction (40).

## 4 IL-33 as immune checkpoint

IL-33 is a pleiotropic cytokine with dual roles, modulating either pro-tumoral or anti-tumoral immune responses within the tumor microenvironment. Its pro-tumor potential is mediated through various mechanisms, including the accumulation of immune-suppressive cells, increased glucose uptake and glycolysis via the upregulation of the membrane glucose transporter 1 on tumor cells, macrophage polarization, and the release of cytokines that promote tumor invasion and migration, such as metalloproteases and TNF-α (41).

Several reports have highlighted IL-33’s potential as an immune checkpoint inhibitor. This concept was first proposed by Kevin Van der Jeught et al. (42). In a colon cancer model, they demonstrated that the anti-tumor effect of anti-PD-1 mAb was enhanced in ST2 knock-out mice. Moreover, they developed a fusion protein consisting of the extracellular part of ST2 and its coreceptor IL-1RAcP, linked by a flexible linker. This protein, known as IL-33trap, was able to neutralize IL-33 in the tumor microenvironment and efficiently reduced ST2-expressing tumor-associated macrophages, thereby limiting the infiltration of immune-suppressive cells (42). The role of IL-33 as an immune checkpoint was further confirmed by Mariana Z. Jovanovic et al. (43). They demonstrated in mouse models of breast and colon cancer an enhanced antitumor effect of anti-PD-1 mAb in ST2 knock-out mice. Interestingly, this effect was replicated with the dual blockade of the IL-33/ST2 and PD-L1/PD-1 axes using antibodies against IL-33 and PD-1. The dual blockade enhanced anti-tumor immunity and reduced tumor growth by boosting NK cells and increasing intratumoral CD8^+^ T cell frequencies and function (43).

## 5 Discussion

As seen in this mini-review, numerous studies highlight the promising antitumor properties of IL-33, whether it is naturally released or externally administered. However, it is crucial to recognize that the impact of IL-33 on tumors can be contingent upon the specific tumor microenvironment. In certain scenarios, IL-33 might paradoxically facilitate tumor growth by fostering the expansion of T regulatory cells and myeloid suppressor cells, both known for their pro-tumoral effects on the immune system (43–45). Consequently, the activity of IL-33 could be envisioned as an amplifier of the pre-existing immune response within the tumor microenvironment.

This dual role underscores the complexity of leveraging IL-33 therapeutically, necessitating further investigation. One potential avenue lies in combining IL-33 with other immunotherapies that stimulate an antitumor immune response. Alternatively, engineering IL-33 could enhance its antitumor efficacy while mitigating its pro-tumoral effects. Hence, IL-33 emerges as a compelling target for cancer immunotherapy, awaiting refined therapeutic strategies to fully harness its considerable potential.

## Author contributions

LA: Conceptualization, Visualization, Writing – original draft, Writing – review & editing. AR: Writing – original draft, Writing – review & editing. ME-H: Writing – original draft, Writing – review & editing. FA: Writing – original draft, Writing – review & editing. PB: Writing – original draft, Writing – review & editing, Conceptualization, Funding acquisition, Supervision, Visualization.
